# Navigating the food environment: Experiences of reduced calorie interventions to manage Type 2 Diabetes Mellitus

**DOI:** 10.1177/13591053241292823

**Published:** 2024-11-21

**Authors:** Rachael McDonnell Murray, Colm Peelo, Fiona Duffy

**Affiliations:** 1 Lynebank Hospital, Scotland; 2 The University of Edinburgh, Scotland; 3 Trinity College Dublin, Ireland

**Keywords:** calorie restriction, diet modification, low-calorie diets, systematic review, T2DM, weight loss

## Abstract

Research into achieving Type 2 Diabetes Mellitus remission through weight loss efforts has grown steadily in the past decade. Most of this research has focused on the effectiveness of weight loss as a method to achieve remission, rather than considering individuals experiences of engaging with such change. This paper aims to review the qualitative research on individuals’ experience of proposed dietary modifications with a weight loss focus. Six databases were searched for qualitative and mixed-methods research studies, and studies were subject to analysis following Thomas and Hardin’s guidelines. The search yielded 2945 results, of which 47 were reviewed in full, and nine were included for analysis. Four analytical themes were identified; variability in support; choosing dietary change; re-negotiating the food relationship; and looking beyond weight loss. Providing tailored nutritional information that is comprehensible and culturally appropriate must be the premise of the interventions offered. Supporting patients to understand their relationship with food and identify meaningful goals beyond weight loss is an important starting point.

## Background

Type 2 Diabetes Mellitus (T2DM) is a complex metabolic disorder characterised by chronic hyperglycaemia that can cause serious medical difficulties if left untreated which can have a significant impact on an individuals quality of life (Carrier & Beverly, 2021; Trikkalinou et al., 2017). Historically, it was considered an irreversible, chronic and progressive condition; however, an accumulating evidence base has revealed that it is possible to achieve remission without pharmacological intervention (Hallberg et al., 2019). There is an ongoing debate regarding the remits of remission (see Taheri, 2021). However, current guidelines within the United Kingdom (UK) consider remission to have been achieved if a recording of haemoglobin A1c (HbA1c) concentration less than 48 mmol/mol is obtained, which must be sustained for 6 months without any pharmacological input (Nagi et al., 2019). Whilst not everyone diagnosed with T2DM is of a higher weight, it is estimated that 90% of adults with T2DM are overweight or obese (Grant et al., 2021). Non-pharmacological treatment approaches specify weight loss as the necessary component to achieve remission for those of any size (Holman et al., 2022). Guidelines recommend between 5% and 10% as a weight loss goal through dietary modification and increased physical activity (National Institute for Health and Care Excellence [NICE], 2014), 2014” is not given in the reference list. Please check and provide the necessary details.].

Modifying diet in response to a diagnosis of T2DM has been well-documented across the literature as an effective way to maintain glycaemic control, and to reduce comorbidities and side effects (Asif, 2014). Whilst these recommendations have always been incorporated into self-management interventions, the shift in using weight loss as a method to achieve T2DM remission has drawn increased attention to all dietary interventions which may be considered effective for long-term weight loss. In a consensus agreement by 131 experts in diabetes treatment, research and remission, agreement was reached that diet should be considered as a primary intervention for T2DM which can result in remission for some, but not all adults. A variation of diets was suggested which ranged from low-fat, whole-food, plant-based diets, avoiding or minimising refined foods, ultra-processed foods and foods with added fats. Calorie reduction was suggested to be achieved by reducing quantity of food intake by reducing portion sizes, or reducing energy density, by using liquid meal replacements or combining approaches (Rosenfeld et al., 2022).

Across the literature there have been advances in how diabetes care is being considered, studied and delivered. There has been an important shift, underpinned by principles of health at every size, towards supporting individuals to engage in health promoting behaviours that acknowledges the complexity of making such lifestyle change (Penney and Kirk, 2015). An important part of the design and delivery of educational programmes on T2DM has prioritised helping individuals to build a healthy relationship with food, by promoting diets rich in nutritional value to nourish their physical health without excluding or eliminating food groups (Hou et al., 2023). With the most recent emphasis on the use of low-calorie diets to support individuals to achieve remission, psychosocial factors must continue to be prioritised alongside medical outcomes of success (Kalra et al., 2018).

We have learned from adjacent literature on dieting amongst those without T2DM, that following a period of restrictive caloric intake, individuals are more at risk for binge eating behaviours, which can result in feelings of guilt, shame and low mood if one is aiming to follow a strict prescribed diet (Craven and Fekete, 2019). This can lead to a vicious cycle of restrictive eating, dieting and bingeing, which often results in short-term weight loss followed by longer-term weight gain (McCuen-Wurst et al., 2018). Despite the multitude of risk factors that make this population vulnerable to developing disordered eating (Sachar et al., 2019; Salvia et al., 2022), this has been neglected and often overlooked in studies (Nieto-Martínez et al., 2017). The limited research which has been conducted has primarily focused on binge eating behaviours (Salvia et al., 2022). However, it is becoming increasingly noted in clinical practice that transdiagnostic or sub-threshold disordered patterns of eating are prevalent among this population (Pekin et al., 2022).

Disordered eating includes behaviours that may be overtly identified as problematic such as restrictive eating, purging, overusing laxatives and excessive exercise. Whilst these behaviours can result in greater physiological harm, they are less likely to be identified in a healthcare assessment. These behaviours are also often overlooked for individuals living in larger bodies. For example, while concern may be expressed for someone who is engaging in restrictive eating living in a smaller body, the same concern may not be afforded to someone in a larger body, who may be praised for engaging in unhealthy eating patterns (Ralph et al., 2022). Prior to an increase in healthcare services adopting weight loss focused interventions, we must consider what has been learned from research exploring participants’ experiences of using low-calorie diets to manage glycaemic control.

Whilst there is a body of literature outlining many of the challenges experienced by those using dietary approaches to achieve weight loss by caloric restriction without a diagnosis of T2DM, the same level of consideration has not been provided for those with T2DM (Hallberg et al., 2019). Those who are of a higher weight and diagnosed with T2DM may present with unique challenges when engaging with weight management interventions because of their additional healthcare needs. For example, they may experience additional pressure by healthcare teams to reduce or restrict foods in order to improve their health outcomes (Taylor, 2019). The aim of this review is to thematically synthesise qualitative data that has been collected on those who have a diagnosis of T2DM and who have made efforts to make a dietary change in response to weight loss-focused advice to manage their diabetes. The objectives of this review are to:

1. Systematically review the literature conducted in this area in order to identify gaps, as well as assess the quality of research conducted to provide clinical and research recommendations.
2. To gain insight into the benefits and challenges that arise for individuals following dietary modification recommendations to manage T2DM.

## Methodology

As the primary aim of this research sought to explore individuals’ experiences making dietary change, a thematic synthesis was chosen as the most appropriate review methodology. This systematic review was prospectively registered on PROSPERO (CRD42022306989) on the 26/01/2022 and is reported according to the ENTREQ statement (Supplemental Material 1; Tong et al., 2012).

### Eligibility criteria

Inclusion criteria: (a) Patients with a diagnosis of Type 2 Diabetes Mellitus, (b) Adults 18 years and older, (c) Studies which focus on the experiences of individuals making dietary change, following weight loss-focused guidance to support the management of T2DM (d) Qualitative research studies and (e) Mixed method studies will be included with only the qualitative component of the research being extracted and analysed, (f) Published papers and grey literature. Exclusion criteria included: (a) Treatment through medication, (b) Treatment through bariatric surgery, (c) Research that is not published in English, (d) Studies which include participants who have not made dietary modifications with weight loss focus and (e) Quantitative research.

### Information sources and search strategy

The following electronic databases were searched on 18/06/2024: Ovid MEDLINE, Ovid Embase, Ovid PsycINFO, EBSCO CINAHL and Web of Science and ProQuest. The author conducted hand searches of reference lists of relevant papers and studies identified in the initial searches.

### Selection process

The database search results were downloaded into Zotero and transferred to Covidence (2023). Duplicates were removed, allowing for the screening of the remaining titles and abstracts against the predefined inclusion criteria. All abstracts and titles were reviewed by the first author (RMcDM), and a second reviewer (CP) independently reviewed 30% of the abstracts and titles. All conflicts were discussed, and an agreement was reached on which studies to consider further. An agreement rating of selected studies was read in full, allowing the full text to be screened against the inclusion/exclusion criteria by one researcher RMcDM. An agreement rating of 99.8% was achieved following this review process. CP independently reviewed 30% of the full texts. All papers were agreed upon for inclusion. Discussions were held for agreed-upon included papers between RMcDM, CP and FD.

### Data items

Study characteristics (study title, author information, country of study, year of publication, methodology, brief description of diet followed and participant characteristics) were extracted manually and exported into a pre-designed table. On-going discussions between RMcDM and FD were held on the data extraction table and presentation. A reflexive log was kept throughout the selection and analysis process.

### Synthesis methods

Analysis was conducted as per Thomas and Harden (2008) guidelines taking a three-step approach; steps 1 and 2 included line-by-line coding to develop descriptive themes; following the identification of similarities and differences between codes to group them into a hierarchical structure; finally, descriptive themes were reviewed to go beyond the data and generate analytical themes. Data from the ‘results’ section were considered participant quotes and author interpretations. Data were extracted to NVivo 11 Plus for analysis (Allsop et al., 2022).

The first author, who has experience in qualitative methodology currently working within a weight management service as a trainee clinical psychologist, read each full-text several times to become familiar with the data. Coding was conducted by the first author (RMcDM). Regular conversations were held with FD (background in Clinical Psychology with expert knowledge of qualitative methodology) and CP (a PhD student with a background in Psychology and Neuroscience) while the coding framework was being developed and when identifying analytical themes. Illustrative quotes were chosen for each analytical theme and sub-theme. Initial descriptive themes were presented at an Eating Disorders and Behaviors Research group to seek feedback, which includes a panel of researchers and individuals with lived experience. Example analysis and coding frame development are presented in Supplemental Material 2.

### Certainty assessment

The Modified version of the Critical Appraisal Skills Programme (CASP) quality assessment tool was employed to review the quality of papers selected for inclusion (CASP, n.d; Long et al., 2020). As it was intended for the findings of this review to provide clinical and research recommendations to inform policy and best practice, the decision was made to use GRADE-CERQual. This tool is employed to review confidence in the evidence of findings from qualitative research (Munthe-Kaas et al., 2018). The first author (RMcDM) used the CASP tool to review all included papers. A second reviewer (CP) reviewed 20% of included papers. Discussions were held between reviewers on choices regarding the quality review.

## Results

### Study selection

A PRISMA flowchart is presented in Supplemental Material 3, which illustrates the various stages of the review.

### Study characteristics

Eleven peer-reviewed papers were included for analysis in this review. Two of the papers drew from the same sample undertaking the same programme (Rehackova et al., 2017, 2020), one of which included a longitudinal follow-up time point. The intensity and frequency of support available varied significantly across studies. Most studies explored participants’ experiences of a structured programme which included a prescribed diet (Brooks et al., 2024; Bynoe et al., 2020; Dhir et al., 2023; Maglalang et al., 2017; Rehackova et al., 2017, 2020, 2022a; Wycherley et al., 2012). There was a mix of dieticians (*n* = 3), multidisciplinary team (*n* = 2), research staff (*n* = 2), ‘service providers’ (*n*−1) and a family practitioner (*n* = 1) who delivered the interventions. For the remaining three studies, there was a variation in healthcare professionals who provided dietary guidance. The frequency and intensity of the programmes varied with some participants being asked to log their food intake daily with ‘ad hoc’ virtual group discussions (Maglalang et al., 2017), to biweekly check-ins either by telephone or in clinic from 12 weeks to 1 year (Brooks et al., 2024; Dhir et al., 2023; Rehackova et al., 2017, 2020, 2022a). Three studies were not associated with delivering a structured intervention. These studies explored participants modifying their diet in response to guidance offered by healthcare professionals. The support and advice offered across these studies varied (Moore et al., 2019; Vijan et al., 2005), with the exception of Webster et al. (2019) where all participants followed the same diet. Blood glucose levels and weight loss was outlined as a measure of success in nine of the studies (Brooks et al., 2024; Bynoe et al., 2020; Dhir et al., 2023; Maglalang et al., 2017; Rehackova et al., 2017, 2020, 2022a; Vijan et al., 2005; Webster et al., 2019), and described in one study (Moore et al., 2019). The characteristics of each study are presented in Table 1 (Supplemental Material 4).

### Quality appraisal

All studies were considered acceptable quality to be included in the analysis of findings (Full CASP review available in Supplemental Material 5). All themes were considered medium confidence or above based on a GRADE-CERQual assessment (See Supplemental Material 6). There were a number of methodological limitations that were pervasive across studies. Only one study discussed the ontological or epistemological position that was being taken. The relationship between the participants and the researchers was only considered in detail in three studies. A paragraph on reflexivity would have been helpful, particularly for studies where the authors were either being funded by or implementing the intervention. This would help to demonstrate purposeful reflexivity on the researcher’s relationship with the data.

### Themes

There were four analytical themes; (1) variability of support, (2) choosing dietary change, (3) re-negotiating the food relationship and (4) beyond weight loss, and seven subthemes that were identified from the analysis.

#### Theme 1: Variability of support

This theme highlights the variability of support that participants were provided when recommended to lose weight through dietary modification and how participants responded to this support.

##### An opportunity for support

For those offered structured programmes, it was perceived as an opportunity for support. This is illustrated in the poignant quote below which evokes a feeling of uncertainty regarding the next steps towards change, if a programme had not been offered:

My parents both died because of complications of diabetes … I lost weight but then I gained it all back and I was starting to get afraid because … I was starting to be at my heaviest again. I said ‘what will I do?’ … I’m deeply grateful for this opportunity to be part of this study. I would want to see my granddaughter grow up and all my other grandchildren grow up and still be with them (Maglalang et al., 2017: 149).

Deep gratitude was expressed by participants who were offered guidance (Brooks et al., 2024; Maglalang et al., 2017; Rehackova et al., 2017; Wycherley et al., 2012). They valued accountability, and frequency of contact as it became a space to problem solve, and overcome challenges experienced by participants. For some there was a recognition that the opportunity for support was not universally available clinically. This resulted in a pressure to do their best in order not to disappoint research staff/sponsors or ‘distort results of the study’ (Rehackova et al., 2017: 1560)

I do recognise the fact that this is a medical study so I’m one of a handful of people that are lucky to actually be on it so to not do as what I’m told would just be silly, stupid (Rehackova et al., 2017: 1560).

##### Tailored guidance

The relationship participants had with those prescribing the diet, and their attunement to participants’ nutritional literacy, ethnicity, culture or socio-economic status, appeared to play an important role in how supported or unsupported participants felt across all studies. This influenced participant’s engagement with programmes, as highlighted in Maglalang et al.’s (2017) research which revealed that over half of the participants stated that the culturally tailored support in terms of materials and staff enhanced their engagement. Access to frequent support, and providing the opportunity to build rapport was noted to be central in reducing barriers in implementing and sustaining healthcare recommendations regarding dietary choices:

The regular check-ups and the fact that you’re being monitored and the fact it’s part of a clinical trial, you can’t just give in because you’re letting other people down as well as yourself. That keeps you honest, and it makes it a lot easier. I would say my chances of being where I am just now without that help would be as much as half (Rehackova et al., 2022a: 9).

For participants offered continuous support to facilitate change, the transition following the end of the programme was noted to be the most significant challenge. Highlighting the loss of a relied upon source of support:

You go from intensive supervision to no supervision at all at the conclusion of the programme. You don’t have regular weigh-ins or anything like that afterwards. The weigh-ins and that sort of thing are incentives during the study. Left to your own devices, you don’t have that to look forward to and tend to let things slide (Wycherley et al., 2012: 636).

Those following a prescribed diet without intensive or frequent guided support experienced additional barriers to making dietary change. Healthcare professionals holding unrealistic expectations for participants without supportive follow-up, or disapproval of chosen dietary modifications, resulted in a rupture of the relationship between participants and their healthcare provider. Some participants felt there was a lack of understanding by those delivering dietary advice on the traditional foods that were central to their community, which also contributed to a distrust in the therapeutic dynamic. It seemed that not having the opportunity to develop rapport with their healthcare provider impacted on the quality of the support that met their individual needs (e.g. Dhir et al., 2023).

See, that is where it comes down to these doctors again. They don’t understand the community or your upbringing … It ain’t got nothing to do with black or white. I am a southerner man. They cook like this all the time (Vijan et al., 2005: 35).

Following recommendations was also a significant financial challenge for some people who felt their healthcare provider failed to consider their socio-economic position:

My wife and I went down and did an inventory of what it would cost to get the dietetic food … we spend about $250 a month now for food for the wife and I It would have been $450 the other way … so we have to buy the cheap stuff (Vijan et al., 2005: 36).

Socio-economic class, or availability of foods were not discussed in studies where the prescribed diet was provided free of charge (Brooks et al., 2024; Bynoe et al., 2020; Rehackova et al., 2017, 2020, 2022a).

#### Theme 2: Choosing dietary change

To improve health outcomes, by managing T2DM, was a driving force for participants making dietary change. For the five studies focused on remission, participants frequently equated weight loss as the method in which they would improve their health and manage their diabetes (Bynoe et al., 2020; Rehackova et al., 2017, 2020, 2022a; Webster et al., 2019). For those wishing to increase physical activity, weight loss was also perceived as a necessary step that would allow them to participate in exercise. Dietary change was thus a method through which some participants felt they could lose weight, and in turn improve their health outcomes. This contrasted with the motivations voiced in the study by Moore et al. (2019), in which participants desired ‘a smaller waist whilst retaining a curvy shape’ (p. 8) rather than to lose weight.

##### Motivation for change

Increasing mobility, increasing energy levels, well-being, to manage their diabetes without the use of medication, and to prolong their life expectancy were motivations identified by participants (Rehackova et al., 2017, 2020, 2022a; Webster et al., 2019; Wycherley et al., 2012). Fear of extreme health consequences that may arise from untreated T2DM heighted the sense of urgency to engage with lifestyle change ‘We can see the complications, there are some people with amputated legs, some people with blindness’ (Moore et al., 2019: 6).

A preference for managing diabetes over pharmacological intervention was attributed to participant’s reluctance to rely on medication for the rest of their lives. For some participants, the side-effects of the medication were experienced as unpleasant. This evoked worry that medication would have a negative impact on their physical health:

When I was first diagnosed with diabetes it was like the end of the world, I had no way out. I’m gonna be living on medication for the rest of my life and all those medications are gonna destroy me from the inside out … (Maglalang et al., 2017: 149).

Many participants noted that they wished to increase their life expectancy in order to spend more time with their family. To live long enough to experience significant life events:

This is captured in one quote by a participant who stated: ‘My mom died of cancer and diabetes, it became personal to me … I want to live more … I have my own two kids now … I want to see their kids graduate high school, and college’ (Maglalang et al., 2017: 149).

##### Building a support network

Beyond the structure and support of healthcare professionals or research staff, perceived support from family members, friends and colleagues played a central role in facilitating participants to make dietary change. A ripple effect occurred once they began to make changes, which influenced the diet and behaviour of those around them (Brooks et al., 2024; Bynoe et al., 2020; Maglalang et al., 2017; Rehackova et al., 2017, 2022a). This was a reciprocal process in which individuals felt supported in their journey by those around them, but also became the motivators and supporters of others making change:

It went from being lonely like you’re doing it all by yourself … I got twenty to thirty people [to use a Fitbit and participate in weekly challenges] … We changed the whole lifestyle of our family cause everybody’s eating healthy (Maglalang et al., 2017: 149).

It was also noted by a number of authors that as participants began to lose weight, compliments received on physical appearance was perceived as support by some participants to continue with their weight loss efforts. For example, comments about weight loss were interpreted as a compliment for most: ‘receiving compliments on appearance was one of many ways participants felt supported during the very low-calorie diet’ (Rehackova et al., 2017: 1560). This appeared to differ depending on gender, weight loss was perceived positively for women, and but negatively for men, ‘it’s sad to hear things “man you looking small, you looking bony. What wrong with you?”’ (Bynoe et al., 2020: 1822). This was also reported by Brooks et al. (2024).

##### Re-negotiating the food relationship

There was a re-negotiation process which occurred for most with regards to their relationship with food. There was an increased awareness and focus on exactly what participants were eating, the quantity, the frequency of meals and snacks and the nutritional value.

##### Theme 3: Navigating the food environment

For those on total food replacement diets, navigating situations where food would be present was given significant consideration by participants. Strategies were employed to facilitate ‘adherence’, which included food removal, avoidance of a variety of situations, hunger management strategies, planning, weighing self and food and accountability by telling others about their diet (Rehackova et al., 2017, 2020; Webster et al., 2019; Wycherley et al., 2012).

For those on the liquid shake diets, portion control became a greater source of stress during the food re-introduction. Which evoked feelings of anxiety and uncertainty about getting it right, ‘I like regimented things, and being told you could only have soups and shakes and so much vegetables per day was spot on for me but then it comes down to “you take control.” I’m not very good at that’ (Rehackova et al., 2020: 956).

This led to some participants adopting compensatory behaviours to further restrict food, following the re-introduction of food. Research captured by follow-up interviews conducted after the programme had finished noted the continued use of highly restrictive calorie counting:

I’ll have to have periods of dieting extremely hard before and after each event to put it down, but I’m prepared for that. I’ve bought myself some Slimfast, which is what I’ve been using this last month. So, every second day I go on to basically consuming 800 calories, and then it means that I can perhaps go up to about 3000 on these events, and it shouldn’t affect me too much (Rehackova et al., 2022: 958).

Feelings of hunger were reported by participants in three of the research studies (Moore et al., 2021; Rehackova et al., 2022; Vijan et al., 2005). For some, hunger was notable during the initial stage of the diet as they began to significantly reduce calories whereas for others, feelings of hunger continued:

The hunger sort of came and went. It didn’t always stay, but the majority I was hungry for the first probably a couple of months. And I thought it would alleviate, and no. There are days I don’t think about food at all, and there are days I get really hungry (Rehackova et al., 2022: 4).

There was also a thread of deprivation experienced by participants. The importance of enjoying food was described, which was phrased in ways that seemed to conflict with the dietary recommendations that they were provided: ‘I eat mostly our food and I enjoy it, I can’t leave it, no matter what the doctor says’ (Moore et al., 2019). It’s interesting that participant’s hopes were to improve their health, and engage in meaningful lives, but as articulated by one participant, being able to enjoy food was an important part of their reason for living: ‘If I’m going to be alive today, I am going to eat what I want. Otherwise, there is no sense in being here if you can’t enjoy something about it’ (Vijan, 2005: 36).

##### The challenges of socialising

Negotiating social situations for all those adopting dietary modifications was a notable challenge. In order to follow strict food rules according to prescribed guidelines, or caloric intake, participants struggled to engage socially in the way they had previously. For those undertaking total food replacement diets, there was a disruption in traditional family routines, or social eating habits which lead to emotional detachment and loneliness (e.g. Brooks et al., 2024; Dhir et al., 2023). One participant in Maglalang et al.’s (2017) study reported self-isolating during mealtimes conflicted with the participant’s cultural Filipino values, in which participation in family meals was reported to be a sign of respect.

Challenges with socialising were common across all studies although the total food replacement diet presented unique challenges as participants were unable to share family dinners or eat in restaurants. Participants noted that refusing food offered by others led to feelings of guilt and discomfort ‘ … you can’t go in people’s house and not take what they offer you, they feel that you think what they have is not good enough’ (Bynoe et al., 2020: 1822).

Being in restaurants where options within the dietary guidelines were not available, became a source of stress rather than a source of relaxation, comfort or enjoyment: ‘Having supper in a restaurant is a problem in the majority of restaurants. It’s just not enjoyable anymore’ (Webster et al., 2019: 2577).

##### Theme 4: Looking beyond weight loss

This theme pushes beyond the delivery and journey participants experienced along their healthcare trajectory to an understanding of the longer-term implications of engaging with a diet to manage their T2DM. It’s worth considering the impact that having a weight loss focus has for participants of these studies. For example, a few studies captured valuable insights when participants began reflecting on themselves and their self-perception in the context of weight change. Participants’ accounts reflected a critical stance towards their eating behaviours, or in some cases towards people living in a larger body.

It was described by Webster et al. (2019) that ‘several participants identified themselves as carbohydrate or sugar addicts’, and gave one illustrative quote of a participant who had tied their perception of themselves as an ‘addict’ to the need to eliminate carbs forever ‘I have come to understand with low carb, that I’m an addict. Carbs are something that I’m probably never going to be able to eat for the rest of my life’ (p. 2576).

Brooks et al. (2024) outlined how participants equated their weight loss as evidence of effectiveness of the intervention; those who felt they were not achieving their weight loss goal attributed this to their own failure to adhere to the intervention.

Meaningful change did go beyond weight loss for many participants. Authors described the benefits of increased well-being, levels of energy, increased self-efficacy, newly acquired cooking skills, noticing levels of hunger and fullness and confidence in their ability to make lifestyle changes (Brooks et al., 2024; Rehackova et al., 2017, 2020; Webster et al., 2019; Wycherley et al., 2012). There were a notable lack of participants’ quotes regarding these changes across the nine studies.

It feels like I’ve had a spring added into my step. I know that sounds really weird, but it’s just given us a lot more confidence, and I feel quite confident now, and I feel quite … I feel more able to face things and more able to cope with things better, but I think it’s because my mind seems to be in a more focused frame of mind. (Rehackova et al., 2020: 959).

Rehackova et al. (2017) provided insight into participant’s appreciation for increased time engaging in activities such as walking, climbing stairs, working in the garden or playing with grandchildren. Becoming more in tune with patterns of comfort eating, or how they respond to stress was also voiced.

## Discussion

As the emerging evidence on remission has changed the landscape of diabetes care, it is crucial that we look towards the studies that have provided insight into what’s helpful and what’s harmful with weight loss approaches. This is the first review to collate all qualitative research which has been conducted to explore individuals’ experiences of dieting with a weight loss focus to manage their T2DM. There were four analytic themes developed that captured how participants responded to guidance offered. Being offered a structured programme provided participants with an opportunity for tailored support and guidance, which influenced participant’s engagement with dietary change. Re-negotiating the food relationship provided insights into how participants navigated life whilst on a diet. The final theme, beyond weight loss, sits separately to the other themes, which provides the space for the consideration of benefits or harms that may arise for participants beyond their weight loss journey.

It was clear that there was a determination for all participants to improve their health outcomes, however, depending on the frequency, and type of support available, making significant changes was much more challenging for some participants than others. Participants were not afforded the same opportunities for support. Healthcare providers who neglected to acknowledge and tailor advice based on an individual’s food preferences, cultural or traditional values and socio-economic circumstances, resulted in an increased difficulty for participants to even make a start towards dietary change. All of these have previously been identified as barriers in making lifestyle changes (Cheng et al., 2016), reinforcing the critical need for all assessments, guidance and interventions to be delivered in a person-centred way. Researchers have shown person-centred care and length of time spent with patients, providing lifestyle guidance, is associated with increased efficacy for managing glycaemic control, increasing activity levels, improving eating patterns and with longer lasting results (Al Harbi et al., 2022; Asmat et al., 2022).

Fears of a reduced quality of life and decreased life expectancy were extreme drivers for some participants to make dietary changes, in particular for those with family members who had a diagnosis of T2DM. These findings are similar to those identified by a systematic review conducted by Harper et al. (2018), who explored people’s experiences of very low-calorie diets for weight loss purposes without T2DM. Participants interpreted being accepted on to an intervention as a ‘last chance’ for support and to make changes for health and well-being. This feeling of urgency may be more pronounced for those with T2DM, which provides context for the sincere gratitude and fear of failing, or disappointing staff expressed by participants provided a structured programme in this review. Individuals engaging in diet modification for weight loss purposes are a highly motivated group of individuals, powerfully expressed by some participants here. Despite this, a large body of literature continues to identify motivation being a significant barrier to change, rather than considering the wider systemic issues that play a central role in individuals’ ability to sustain such changes (Anderson Steeves et al., 2014). Drastic public health measures must be taken through legislative and policy change in order to shift the responsibility from individual, to the food environment (Walls et al., 2011).

Some of the concerns raised here reflect the growing understanding of the harms dieting can have on an individual’s self-perception, social network and eating behaviours (Robinson et al., 2020). Participant’s experiences of deprivation pulls into question the recommendations of extending the liquid shake diet phase or introducing a ‘rescue plan’ (Marples et al., 2022), if individuals struggle to lose weight, or are beginning to re-gain weight. Deprivation of social connection as a result of dietary choices was also identified from participant accounts. Navigating the food environment resulted in an oscillation of guilt for refusing food and declining social invitations, frustration for not being able to eat out and an acceptance of needing to impose social isolation to follow dietary requirements. Vanstone et al. (2017) highlights the social significance of food being a barrier to individuals making dietary modification which resonates with participant’s experiences presented here. It is noticeable that throughout every theme there is an interpersonal element, building a relationship with a healthcare provider, seeking support from friends and family, losing weight to engage in valued activities and prolonging life to be around for significant family life events. However, the imposed social isolation that dieting culture often creates, conflicts with many of the participant’s hopes to increase quality time with family and friends.

The final theme considers the necessary components that often underpin sustained health-promoting behaviours over time. Looking beyond weight loss and supporting individuals to identify goals that are aligned with what they value may support longer-term behavioural change (Greaves et al., 2017). In this review, limited data was available exploring people’s individual goals or progress made towards achieving them. Rather than outcome measures of BMI being considered as success, perhaps a re-focus of building a healthy relationship with food, with oneself and engaging in valued activities may also facilitate a shift in attention towards sustainable health-promoting behaviours over time, rather than restrictive eating strategies (Fink et al., 2019).

### Strengths and limitations

There are a number of strengths and limitations to this review that must be acknowledged. As nearly half of the studies were of mixed methods, direct participant quotes were limited. Whilst this is a valuable finding in and of itself, analytical themes must be considered with this caveat in mind. There was a variability in weight loss efforts by participants and guidance provided. The decision was made to include all studies which asked participants about their experience of weight loss focused dietary modification, because of the limited available evidence for one specific diet. This appeared to be directly influenced by recent evidence on the use of weigh-loss programmes to support remission. Most programmes included here were available because of research funded studies and were not routinely offered as part of clinical care, which may have influenced a more favourable view of their experience.

### Conclusion

The main strength of this review is the timely collection and review of the available research conducted exploring people’s experiences of energy restrictive diets. Given this approach is being rolled out on a national level within the United Kingdom, hearing from the voices of participants helpful in understanding the main strengths, and potential harms of this approach.

## Supplemental Material

sj-docx-1-hpq-10.1177_13591053241292823 – Supplemental material for Navigating the food environment: Experiences of reduced calorie interventions to manage Type 2 Diabetes Mellitus Supplemental material, sj-docx-1-hpq-10.1177_13591053241292823 for Navigating the food environment: Experiences of reduced calorie interventions to manage Type 2 Diabetes Mellitus by Rachael McDonnell Murray, Colm Peelo and Fiona Duffy in Journal of Health Psychology

sj-docx-2-hpq-10.1177_13591053241292823 – Supplemental material for Navigating the food environment: Experiences of reduced calorie interventions to manage Type 2 Diabetes Mellitus Supplemental material, sj-docx-2-hpq-10.1177_13591053241292823 for Navigating the food environment: Experiences of reduced calorie interventions to manage Type 2 Diabetes Mellitus by Rachael McDonnell Murray, Colm Peelo and Fiona Duffy in Journal of Health Psychology

sj-docx-3-hpq-10.1177_13591053241292823 – Supplemental material for Navigating the food environment: Experiences of reduced calorie interventions to manage Type 2 Diabetes Mellitus Supplemental material, sj-docx-3-hpq-10.1177_13591053241292823 for Navigating the food environment: Experiences of reduced calorie interventions to manage Type 2 Diabetes Mellitus by Rachael McDonnell Murray, Colm Peelo and Fiona Duffy in Journal of Health Psychology

sj-docx-4-hpq-10.1177_13591053241292823 – Supplemental material for Navigating the food environment: Experiences of reduced calorie interventions to manage Type 2 Diabetes Mellitus Supplemental material, sj-docx-4-hpq-10.1177_13591053241292823 for Navigating the food environment: Experiences of reduced calorie interventions to manage Type 2 Diabetes Mellitus by Rachael McDonnell Murray, Colm Peelo and Fiona Duffy in Journal of Health Psychology

sj-docx-5-hpq-10.1177_13591053241292823 – Supplemental material for Navigating the food environment: Experiences of reduced calorie interventions to manage Type 2 Diabetes Mellitus Supplemental material, sj-docx-5-hpq-10.1177_13591053241292823 for Navigating the food environment: Experiences of reduced calorie interventions to manage Type 2 Diabetes Mellitus by Rachael McDonnell Murray, Colm Peelo and Fiona Duffy in Journal of Health Psychology

sj-docx-6-hpq-10.1177_13591053241292823 – Supplemental material for Navigating the food environment: Experiences of reduced calorie interventions to manage Type 2 Diabetes Mellitus Supplemental material, sj-docx-6-hpq-10.1177_13591053241292823 for Navigating the food environment: Experiences of reduced calorie interventions to manage Type 2 Diabetes Mellitus by Rachael McDonnell Murray, Colm Peelo and Fiona Duffy in Journal of Health Psychology
