# Supplementary material for: Navigating the food environment: Experiences of reduced calorie interventions to manage Type 2 Diabetes Mellitus
Source: J Health Psychol. 2024 Nov 21;30(10):2429–42. doi: 10.1177/13591053241292823 (PMC12381385; doi:10.1177/13591053241292823)
Supplement: sj-docx-1-hpq-10.1177_13591053241292823 – Supplemental material for Navigating the food environment: Experiences of reduced calorie interventions to manage Type 2 Diabetes Mellitus [file sj-docx-1-hpq-10.1177_13591053241292823.docx]

Step 1: Generating initial codes

This step was conducted by first author. Once a list was generated, this was reviewed by the third author. Discussions were held on direction being taken, and considering any bias that may be arising in the identification of codes and initial considerations for descriptive themes.

**Descriptive theme ideas**

1^st^ – refers to direct patient quote

2^nd^ – refers to authors interpretation

Motivation to engage with programme

- ‘I want to live more’ (1^st^): To increase life expectancy to be healthy to watch grandkids grow older/ regain control over their heatlh
- To decrease medication use for diabetes control (1^st^)
- To lose weight (2^nd^)
- Hope, guidance and support
- To achieve T2DM remission (2^nd^

Adherence to the suggested dietary behaviour

Facilitators

- Tailored diet plan (1^st^)
- Collaborative support from staff (1^st^)
- Staff providing culturally relevant dietary advice (1^st^)
- Peer support from others on the programme (1^st^)2^nd^ – sense that participants would have liked more
- Comparison between others on the programme
- Motivation as result of weight loss (1^st^)
- Support from family changing dietary behaviours (1^st^) - and also from family members who complete the cooking
- Social support (1^st^, 2^nd^)
- Support from friends changing dietary behaviour (1^st^)
- Receiving compliments for losing weight – motivation to continue going (1^st^)
- Regular check in’s and monitoring progress (1^st^) - Incentive not to disappoint staff/ feeling grateful for the opportunity to be part of a study.
- Opportunity to take part in a study/programme (1^st^2^nd^)
- Specific strategies to change eating habits
- Strategies – tracking food intake (1^st^)2^nd^ – found to be a tedious task
- Strategies – coping with cravings (1^st^) - distraction/avoidance of situations that would often be paired with snacking such as watching television.
- Strategies – self regulatory skills (1^st^)2^nd^ – behaviour regulation skills
- Strategies – noticing eating habits (1^st^)

Challenges

- Navigating social engagement with friends (1^st^) - both in the sense of eating out for meals/navigating food at special occasions/responding to unhelpful comments from friends/ Change in behaviour at mealtimes with family
- How weight loss is perceived (1^st^)
- Staff not providing culturally relevant advice (1^st^)
- The cost of food (1^st^)
- Boredom with repetition of meals (1^st^) - also rigidity of the diet (this was both seen as a positive and a negative thing)
- Keeping track of calories/food intake (1^st^)
- Hunger (1^st^) 2^nd^
- Unclear dietary advice (1^st^)
- Fatigue (1^st^)

Following the intervention

Benefits/ positive aspects experienced by participants

- Self-confidence/self-belief (1^st^) - feeling in control of being able to manage their diabetes. Confidence that they can achieve a healthier way of living. (One negative example of an individual weighing every morning as a sense of control).
- Feeling healthier (1^st^) - Increased energy/ reduced pain
- T2DM remission (1^st^)
- Increased confidence
- Weight loss (Beyond?)
- Change in eating behaviours
- Change in relationship with food

Negative aspects following intervention

- Feeling it is not sustainable (1^st^) (and one example of a study where they felt it was unsustainable)
- Re-introducing food was perceived as most challenging aspect

**Reflection: What we’re not hearing**

- Disappointment if participants did not achieve remission? How does this feel, what are they offered, what happens next?
- Well-being? Are individuals enjoying their food/ increased self-compassion/ emotional regulation – emotional eating?

Step 2. Initial codes and development of descriptive themes

| **Motivation to engage with the programme** | **Adherence to suggested dietary modification** | | **Benefits experienced following changes in eating behaviours** |
| --- | --- | --- | --- |
|  | Facilitators | Challenges |  |
| - ‘I want to live more’ increase life expectancy  - experience of family member experiencing diabetes complications  -failed past attempts to make change  - live to see grandchildren grow up  -diabetes remission  - weight loss  - increase quality of life  - gratitude for being offered an opportunity  - hope for increase mobility | - Feeling healthier  - simplicity of diet plan  -enhanced engagement through culturally relevant material  - tailored approach to diet changes  - collaborative approach with staff  -encouragement by staff, family, friends  - feeling heard/understood by staff  - use of technology to track and support diet change  - sharing knowledge with friends/family  - ripple effect of lifestyle change on others  - involvement in research study  - accountability  -rapid weight loss  - not wanting to disappoint others  - employment of strategies to reduce cravings  - avoidance of situations where food may be tempting  - structured food plan  - feedback on progress  - avoidance of restaurants  - compensation using shakes  - building trust and rapport with healthcare provider | - Navigating social situations  -boredom of diet plan  - comfort eating  -situations with tempting food on display  - the smell of tasty food  - cost of the diet  - feeling hungry  - engagement at social occasions  - communication with health care staff  - rigid food plan  - feeling rude/guilty for not accepting offers of food  -reduce enjoyment of food  - confusion about dietary advice  -frustration at having to limit portion sizes or cut out foods  -criticism from others about dietary changes  -shame/awkwardness in social situations  - challenges with socialising  -judgement from others about diet  - barriers in accessing certain foods  - fatigue | - Self-confidence/self-belief in ability to make lifestyle changes  - taking a proactive stance  - improvement in physical fitness  - perceived increase of energy  - tuning into hunger feelings  - dig in the garden as a result of increased energy  - feeling of control  - accomplishment  - Achieving T2DM remission    Benefits |

Step 3. Progression of codes and development of analytical themes

All authors participated in reviewing participant quotes and the development of analytical themes. This involved forward and back discussions over the course of a number of weeks over e-mail and verbally. Codes were used to identify sub-themes. Once sub-themes were identified, the framework below to review all manuscripts to identify relevant participant quotes.

**Theme 1. Motivation for making dietary change**

Subthemes

Fears of reduced quality of life and life-expectancy

External factors and missed opportunity for support

To reduce or stop medication use

**Theme 2. Perceived support and supporting others**

Subthemes

Perceived social support by others

Personalised care being offered

The delivery of care

**Theme 3. Re-negotiating the food relationship**

Subthemes

Navigating the food environment

The challenges of socializing after making dietary changes

Feelings of deprivation

**Theme 4. Beyond weight loss?**

Subthemes

Meaningful change beyond weight loss

Negative attitudes expressed towards weight gain
