# Supplementary material for: Navigating the food environment: Experiences of reduced calorie interventions to manage Type 2 Diabetes Mellitus
Source: J Health Psychol. 2024 Nov 21;30(10):2429–42. doi: 10.1177/13591053241292823 (PMC12381385; doi:10.1177/13591053241292823)
Supplement: sj-docx-2-hpq-10.1177_13591053241292823 – Supplemental material for Navigating the food environment: Experiences of reduced calorie interventions to manage Type 2 Diabetes Mellitus [file sj-docx-2-hpq-10.1177_13591053241292823.docx]

Records identified through database searching (n=2,945)

EMBASE (n=2510)

Medline (n=256)

CINAHL (n=134)

PsychInfo (n=22)

PROQUEST (n=248)

Web of Science (n=258)

(Duplicated removed n= 423)

(n = 1044 ) + 675&329 medrix?

## Identification

Additional records identified through other sources
(n=6)

(3 duplicates)

## Screening

Records after duplicates removed

(n= 2,747)

Records screened by abstract and title
(n = 2,747)

Records excluded (n = 2,700)

## Eligibility

Full-text articles assessed for eligibility
(n = 49)

Full-text articles excluded (n = 38)

Wrong study design (n=17)

Wrong intervention (n=18)

Full article has not been written (n=2)

Wrong patient population (n=1)

## Included

Papers included in qualitative synthesis

(n= 11)
