# Supplementary material for: Navigating the food environment: Experiences of reduced calorie interventions to manage Type 2 Diabetes Mellitus
Source: J Health Psychol. 2024 Nov 21;30(10):2429–42. doi: 10.1177/13591053241292823 (PMC12381385; doi:10.1177/13591053241292823)
Supplement: sj-docx-3-hpq-10.1177_13591053241292823 – Supplemental material for Navigating the food environment: Experiences of reduced calorie interventions to manage Type 2 Diabetes Mellitus [file sj-docx-3-hpq-10.1177_13591053241292823.docx]

**Table 1. Studies summarised**

| **Authors** | **Conflict of interest** | **Country** | **Study design and data collection points** | **Programme** | **Sample size** | **Demographics** |
| --- | --- | --- | --- | --- | --- | --- |
| Brooks et al. (2024) | No | United Kingdom | Qualitative study (semi-structured interviews following intervention) | Remotely supported energy formula-based total diet replacement (TDR) or an intermittent low-energy diets (ILED). | 20 | Eleven women and 9 men. Ethnicity 16= white British 2= other black; 1 Indian; 1 Caribbean. |
| Bynoe et al. (2020) | No | Barbados | Mixed method study (semi-structured interviews at baseline, 8 weeks and 5 months) | An 8-week total food replacement diet, consisting of 800 kcal a day. Followed by 4-week food re-introduced phase. Facilitated by a dietician. | 25 | Ten men and 15 women.  Ethnicity = 88% classified themselves as black ethnicity, 8% as mixed, 4% as white. |
| Dhir et al. (2023) | No | United Kingdom | Qualitative study (semi-structured interviews | A 12-week total diet replacement phase (estimated 900 kcal/day) followed by a 6-week stepped food reintroduction (FR) phase and weight maintenance (WM) support until programme end. | 12 | Seven females, and five males. Ethnicity = Pakistani (n=6), Indian (including Gujarati and Hindu) (n=4) and Bangladeshi (n=2) and there were slightly more females (n=7) than males (n=5) |
| Maglalang et al. (2017) | No | United States of America | Mixed methods study (semi-structured interviews at baseline, 3-month and 6-months) | Three-month weight-loss lifestyle intervention, self-report food/calorie intake and weight using the Fitbit diary app. Goals of the intervention include weight reduction of at least 7 percent through a healthy low-calorie, low-fat diet and to engage in at least 150 minutes per week. Followed by three-month maintenance phase. Facilitated by research staff. | 45 | 62% of the sample were female.  Ethnicity = all reported as Filipino.  . |
| Rehackova, et al. (2017) | Yes | United Kingdom | Qualitative study (semi-structured interviews before and after completion of the programme). | An 8-week total food replacement diet, consisting of 800 kcal a day. Facilitated by a healthcare team. | 18 | Seven women and eleven men. Ethnicity = not reported. |
| Rehackova1 et al. (2020) | Yes | United Kingdom | Qualitative study (semi-structured interviews at three time points, longitudinal follow-up) | A 12-week total food replacement diet, consisting of 800 kcal a day. Followed by food re-introduction and maintenance phase for up to 2 years**.** Facilitated by a healthcare team. | 11 | Seven men, four women. Ethnicity = not reported. |
| Rehackova1 et al. (2022) | Yes | United Kingdom | Qualitative study (semi-structured interviews before, during the diet, 2 weeks into the food replacement phase and 1 year from the initial interview) | A 12-week total food replacement diet, consisting of 825– 853 kcal/day for 12 weeks. Followed by food re-introduction phase and weight loss maintenance for up to 2 years**.** | 34 | 18 women and 16 men. Ethnicity = all were of white ethnic background. |
| Webster et al. (2019)  . | No | South Africa | Mixed methods study (Semi-structured interviews time point not specified). | Self-selected, low carbohydrate, high fat diet. Average intake was 1,794kcal day. Carbohydrate intake was between 20 and 50 g/d (very low) for 10 participants, between 50 and 115 g/d (low) for 17 participants and was 142 g/day (moderate) for one participant. | 28 | 14 men and 14 women  Ethnicity = Not reported. |
| Wycherley et al. (2012) | No | Australia | Mixed methods (One year following completion of the programme) | A moderately energy-restricted prescriptive diet of 6000 kJ⁄ day 7000 kJ⁄ a day. Facilitated by a dietician. | 30 | 22 males, and 8 females  Ethnicity = not reported. |
| Moore, et al. (2019) | No | United Kingdom | Qualitative study (focus groups, one time point) | Variability of dietary recommendations being followed. | 41 | 27 female, 14 male  Ethnicity = 18 participants were Black African, 22 participants were Black Caribbean. |
| Vijan et al. (2005) | No | United States of America | Mixed methods study (focus groups, one time point) | A diet with sugar, fat, and calorie reduction aimed at weight loss. Varied dietary pattern across participants and prescriber. | 6–12 | 97% of the sample were male.  Ethnicity: 88% white (sub-urban site); 92% African-American (urban site). |
