# Supplementary material for: Navigating the food environment: Experiences of reduced calorie interventions to manage Type 2 Diabetes Mellitus
Source: J Health Psychol. 2024 Nov 21;30(10):2429–42. doi: 10.1177/13591053241292823 (PMC12381385; doi:10.1177/13591053241292823)
Supplement: sj-docx-4-hpq-10.1177_13591053241292823 – Supplemental material for Navigating the food environment: Experiences of reduced calorie interventions to manage Type 2 Diabetes Mellitus [file sj-docx-4-hpq-10.1177_13591053241292823.docx]

2. CASP

| Authors | Question | | | | | | | | | |
| --- | --- | --- | --- | --- | --- | --- | --- | --- | --- | --- |
|  | 1. | 2. | 3. | 4. | 5. | 6. | 7. | 8. | 9. | 10. |
| Dhir et al. (2023) | Yes | Yes | Yes | Yes | Yes | No | Yes | Yes | Yes | Yes |
| Bynoe et al. (2020) | Yes | Yes | Somehwat | Yes | Somewhat | No | Somewhat | Uncertain | Somewhat | No |
| Maglalang et al.(2017) | Yes | Yes | Uncertain | Yes | Yes | Somewhat | Somewhat | Uncertain | Yes | Somewhat |
| Rehackova et al (2017) | Yes | Yes | Yes | Yes | Yes | Uncertain | Yes | Yes | Yes | Somewhat |
| Rehackova et al. (2020) | Yes | Yes | Yes | Somewhat | Yes | Somewhat | Somewhat | Yes | Yes | Uncertain |
| Rehackova et al. (2022) | Yes | Yes | Yes | Yes | Yes | Somewhat | Yes | Yes | Yes | Yes |
| Webster et al. (2019) | Yes | Yes | Yes | Yes | Yes | Uncertain | Yes | Yes | Yes | No |
| Wycherley et al. (2012) | Yes | Yes | Yes | Yes | Yes | Yes | Yes | Yes | Yes | No |
| Vijan et al. (2005) | Yes | Yes | Yes | Yes | Yes | Yes | Yes | Yes | Somewhat | No |
| Moore et al. (2019) | Yes | Yes | Yes | Yes | Yes | Yes | Yes | Yes | Yes | Yes |
| Brooks et al. (2024) | Yes | Yes | Yes | Yes | Yes | Somewhat | Yes | Yes | Yes | No |
