# Supplementary material for: Navigating the food environment: Experiences of reduced calorie interventions to manage Type 2 Diabetes Mellitus
Source: J Health Psychol. 2024 Nov 21;30(10):2429–42. doi: 10.1177/13591053241292823 (PMC12381385; doi:10.1177/13591053241292823)
Supplement: sj-docx-5-hpq-10.1177_13591053241292823 – Supplemental material for Navigating the food environment: Experiences of reduced calorie interventions to manage Type 2 Diabetes Mellitus [file sj-docx-5-hpq-10.1177_13591053241292823.docx]

**GRADE-CERQual**

| Review finding | Contributing papers | Methodological limitations | Coherence | Adequacy | Relevance | CERQual assessment |
| --- | --- | --- | --- | --- | --- | --- |
| Theme 1. Variability of support |  |  |  |  |  |  |
| Tailored guidance | Maglalang et al. (2017)  Rehackova et al. (2017)  Rehackova et al. (2022)  Wycherley et al. (2012)  Brooks et al. (2024)  Dhir et al. (2023) | Minor concerns. | No or very minor concerns | Minor concerns | No or very minor concerns | High confidence |
| An opportunity for support | Brook’s et al. (2024)  Maglalang et al. (2017)  Rehackova et al. (2017)  Rehackova et al. (2022)  Wycherley et al. (2012)  Brooks et al. (2024)  Dhir et al. (2023) | Minor concerns. | Minor concerns | Moderate concerns | No or very minor concerns regarding relevance | Moderate confidence |
|  |  |  |  |  |  |  |
| Theme 2. Choosing dietary change |  |  |  |  |  |  |
| Motivation for change | Rehackova et al. (2017)  Vijan et al. (2005)  Rehackova et al. (2020)  Maglalang et al. (2017)  Brooks et al. (2024)  Dhir et al. (2023) | Minor concern regarding two studies. | Minor concerns | Minor concerns | No or very minor concerns | Moderate confidence |
| Building a support network | Vijan et al. (2005)  Rehackova et al. (2020)  Rehackova et al. (2017)  Maglalang et al. (2017)  Brooks et al. (2024)  Dhir et al. (2023) | Minor concerns. | No or very minor concerns | Minor concerns | Minor concerns | High confidence |
|  |  |  |  |  |  |  |
| Theme 3. Re-negotiating the food relationship |  |  |  |  |  |  |
|  | Maglalang et al. (2017)  Vijan et al. (2005)  Rehackova et al. (2017)  Rehackova et al. (2020)  Wycherley et al. (2011) | Minor concerns. | Minor concerns | Minor concerns | No or very minor concerns | High confidence |
| Navigating the food environment | Vijan et al. (2005)  Rehackova et al. (2020)  Rehackova et al. (2017)  Webster et al. (2019)  Moore et al. (2019)    Bynoe et al. (2020)  Brooks et al. (2024)  Dhir et al. (2023) | Minor concerns. | Minor concerns | No or very minor concerns | No or very minor concerns | High confidence |
| Challenges in socialising | Rehackova et al. (2017)  Vijan et al. (2005)  Moore et al.(2019)  Webster et al. (2019)  Rehackova et al. (2022)  Brooks et al. (2024)  Dhir et al. (2023) | Minor concerns | Minor concerns | Minor concerns of adequacy of data. | No or very minor concerns regarding relevance | High confidence |
| Theme 4. |  |  |  |  |  |  |
| Looking beyond weight loss | Rehackova et al. (2017)  Rehackova et al. (2020)  Webster et al. (2019)  Wycherley et al. (2012)  Moore et al. (2019)  Bynoe et al. (2020)  Brooks et al. (2024)  Dhir et al. (2023) | Minor concerns. | Minor concerns.  (some concerns about the fit between the data from primary studies and the review finding) | Moderate concerns (three studies which together offer moderately rich data, one study offered thing data) | No or very minor concerns regarding relevance | Moderate confidence (concerns regarding the coherence of the review finding, and |
